# In the Eye of the Beholder: Using Photography to Teach Gerontology

**DOI:** 10.1093/geroni/igab046.2798

**Published:** 2021-12-17

**Authors:** Kristina Hash, Matthias Naleppa, Anissa Rogers

**Affiliations:** 1 West Virginia University, morgantown, West Virginia, United States; 2 Radford University, Radford, Virginia, United States; 3 CSUSB, Palm Desert, California, United States

## Abstract

Due to the widespread access to smart phones and similar technology, photography and photographic images have become an ever-present part of contemporary social life. Photographic methods are also growing in their use in higher education pedagogy. As a specific application, photography can be a powerful tool to educate students about aging processes and issues that impact older adults. This poster will explore the use of photographic methods and tools to teach and integrate aging-related concepts into gerontology and social work courses, at both undergraduate and graduate levels. Specifically, the use of digital storytelling, photo mapping, photo voice, and photo therapy will be highlighted. Descriptions of and consideration for assignments along with example student projects will be displayed and discussed as will other potential projects and uses of photographic methods. Attention will be paid to how photographic methods can help students explore the diversity and intersection of individual characteristics and experiences with the aging process and how intersectional identities can influence, and be influenced by, aging and external factors and processes.

